# Bat Ecology and Microbiome of the Gut: A Narrative Review of Associated Potentials in Emerging and Zoonotic Diseases

**DOI:** 10.3390/ani14203043

**Published:** 2024-10-21

**Authors:** Emanuela Bazzoni, Carla Cacciotto, Rosanna Zobba, Marco Pittau, Vito Martella, Alberto Alberti

**Affiliations:** 1 Dipartimento di Medicina Veterinaria, Università degli Studi di Sassari, 07100 Sassari, Italy; e.bazzoni2@phd.uniss.it (E.B.); zobba@uniss.it (R.Z.); pittau@uniss.it (M.P.); 2 Mediterranean Center for Disease Control, 07100 Sassari, Italy; 3 Department of Veterinary Medicine, University Aldo Moro of Bari, 70010 Bari, Italy; vito.martella@uniba.it; 4 Department of Pharmacology and Toxicology, University of Veterinary Medicine, 1078 Budapest, Hungary

**Keywords:** *Chiroptera*, spillover, microbiome, pathogens, zoonotic diseases

## Abstract

In this review, we tentatively tried to connect the most recent findings on the bat microbiome and to investigate on their microbial communities, that may vary even in conspecific hosts and are influenced by host physiology, feeding behavior and diet, social interactions, but also by habitat diversity and climate change. From a conservation perspective, understanding the potentially negative and indirect effects of habitat destruction on animal microbiota can also play a crucial role in the conservation and management of the host itself. According to the One Health concept, which recognizes an interdependence between humans, animals, and the environment, bat microbiota represents an indicator of host and environmental health, besides allowing for evaluation of the risk of emerging infectious diseases. We noticed that a growing number of studies suggest that animal microbiota may respond in various ways to changes in land use, particularly when such changes lead to altered or deficient food resources. We have highlighted that the current literature is strongly focused on the initial phase of investigating the microbial communities found in *Chiroptera* from various habitats. However, there are gaps in effectively assessing the impacts of pathogens and microbial communities in general in animal conservation, veterinary, and public health. A deeper understanding of bat microbiomes is paramount to the implementation of correct habitat and host management and to the development of effective surveillance protocols worldwide.

## Simple Summary

This work provides an overview on the impact of bats’ diet on their intestinal microorganism community and its influence on potential pathogenicity. Human expansion generates natural habitat alterations, which force bats to seek alternative sites, leading to contact with other animals, including humans. Exploring the relationship between the dietary habits of bats and the variety of their microbiome could contribute elucidating the potential role of bats as reservoirs of multidrug-resistant microorganisms and their implications in the dissemination of mutating viruses and antibiotic-resistant bacteria in the environment and possibly in their transmission to human and to domestic and wild animals.

## 1. Introduction

*Chiroptera* (Blumenbach 1779) is a mammalian order including animals commonly known as bats, second to *Rodentia* in species richness and including about 20% of mammalian species diversity [1,2,3,4,5].

Their ability to fly allowed for bats to colonize almost the entire Earth’s surface, except for polar and circumpolar regions and some remote oceanic islands. Furthermore, despite their small size, bats can cover long distances by flying at night, this resulting in seasonal migrations and in a dynamic use of habitats. In tropical zones, bats have reached the highest diversity, where they are, in absolute terms, the most abundant order of mammals [6].

The ecological roles of bats in maintaining the balance of ecosystems are remarkable, and bats can be fundamental to many ecological processes such as pest control, soil enrichment, and pollination of plants consumed as food by humans and other animals [7]. Also, they play a role in dispersion of pioneer plants [8] and in the creation and maintenance of “nuclei of regeneration” in tropical rainforests [9]. Moreover, bat guano produced in caves used as resting sites by many gregarious species is considered one of the most valuable forms of fertilizer [10]. Furthermore, fruit-eating bats represent a protein source for rural communities in several southeast Asian countries and Pacific Ocean islands where they are considered a delicacy [11]. True vampire bats (subfamily *Desmodontinae*) are a target of scientific research due to the presence of desmoteplase, a protein released in their saliva with anticoagulant properties with potential applications in ischemia treatment and prevention [12].

Notably, bats host an everchanging and rich viral community that does not associate with disease status. Bats’ tolerance to viral infections relies on different mechanisms, such as the activation of their innate immune system in unstimulated tissues and metabolic adaptations to flight [13]. The constitutive production of 3 interferon (IFN)-α limits viral replication reducing the need for high antibody titer production and affinity maturation. Furthermore, genome repair pathways were selected in bats to minimize DNA damage due to the high metabolic rates associated with flight, also inhibiting viral modulation activity. Lastly, genome stability together with low telomerase activity have been related to remarkably good aging and resistance to cancer [14,15].

The bat microbial genetic pool includes multi-hosts and/or potentially zoonotic agents. These microbial communities are usually enzootic due to the scarce interaction of bats with human communities and domestic animals in natural habitats [2,7]. Along with the Industrial Revolution, the human population has constantly increased and fueled urbanization, agriculture, and anthropogenic modification of habitats. In turn, human encroachment and loss of habitat have pushed bats to approach urban areas promoting interaction with other animals and the consequent emergence and expansion of new potentially harmful zoonotic diseases [16].

Indeed, the role of bats as reservoirs for microbial agents has been increasingly recognized over the last century, with a major focus on their contribution in spreading viruses and bacteria that can be transmitted to other animal species, including humans [2]. In fact, bats are natural reservoir of several epidemiologically relevant viruses, such as *Filoviridae*, *Henipavirus*, *Lyssavirus*, *Flaviridae*, and *Coronavirus*, which in at least three cases managed to jump to humans (SARS-CoV-1, MERS-CoV, and the pandemic due to SARS-CoV-2) [17]. Many worldwide-distributed bat species transmit rabies virus through bites [18]; Ebola viruses have been isolated in at least three African species of *Pteropodidae* [19]; in 2007, the Marburg virus was identified in specimens of *Rousettus aegyptiacus* [20]. In Asia, some recent studies have highlighted the presence of *Lyssavirus* and *Henipavirus* (Hendra and Nipah viruses) in *Pteropus* spp. [21,22].

Similarly, high-richness bacterial and fungal communities have been described in bats, and their contribution to zoonotic outbreaks and transmission to multiple animal species have been only partially investigated [23]. Many drug-resistant bacterial and fungal species have been identified as common residents in bat mucosae, but the possible role of bats in the transmission of these pathogens to domestic animals and humans must be fully elucidated [24,25,26].

Based on the literature, this review attempts to present comprehensive data on the relationship between the different *Chiroptera* dietary habits and their specific microbial communities.

## 2. Literature Review Process

**Figure 1:**
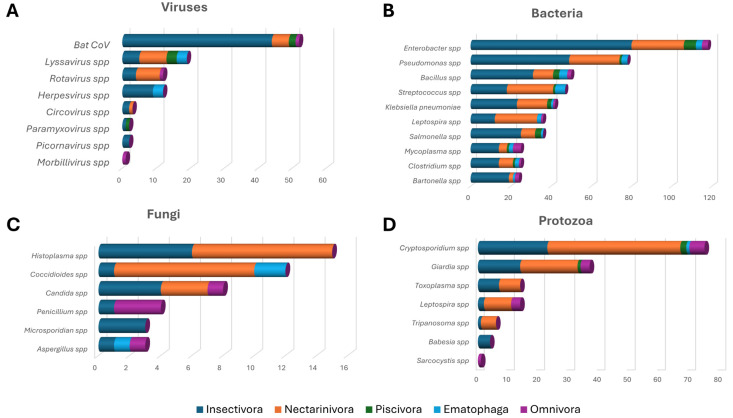
Viral (**A**), bacterial (**B**), fungal (**C**), and protozoan (**D**) infectious agents mostly reported in the literature, worldwide. Infectious agents are grouped according to bats dietary ecology.

**Figure 2:**
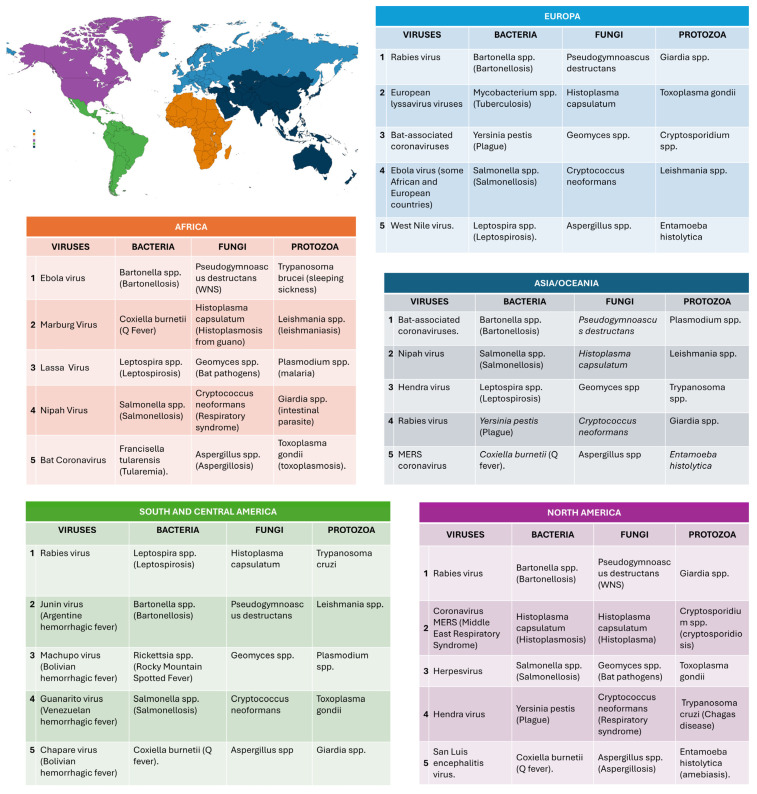
Viral, bacterial, fungal, and protozoan infectious agents mostly reported in the literature and grouped according to continent.

Based on an analysis of the dedicated literature, over 100 microorganisms for which bats play a role as potential reservoirs (viruses, bacteria, fungi, and protozoa) were identified. In addition, studies on serology, microbiota screening, overviews of the interaction with microorganisms in the microbiota, mechanisms of resistance to infection, and the diet-dependent microbiome were taken into account. Screening by title and abstract was performed to remove records that did not describe specific investigations on the bat microbiome or diet (reviews, in vitro and in vivo studies, etc.). Then, the full texts of the remaining records were retrieved to assess their eligibility. Publications that analyzed the human and other animal microbiomes were included in the database when deemed notable and relevant. Preprints were included. The most significant search engines for worldwide publications were https://scholar.google.com/, https://www.researchgate.net/, and https://www.google.it/, and to reach the most prestigious journals and sites with the most recent publications, https://pubmed.ncbi.nlm.nih.gov/, https://www.sciencedirect.com/, and https://www.academia.edu/ were used (Last access date was 20 August 2024). Search queries were “bat microbiome and serology”, “bat microbiota”, “microorganisms in bat microbiota”, “bat microbiota and infection resistance”, “diet-dependent bat microbiota”, “microorganisms in bats and immune resistance”, “bat microbiome and longevity”, “bat resistance to infections”, “bat cancer resistance”, “one health and bat microbiome”, “bat demonization”, and “importance of microbiota in mammals”. Data were then categorized by cross-referencing species and diet, reporting the author, the title of the research, and the microorganisms. An extensive excel database was created with information on the viral, bacterial, fungal, and protozoan microbiome for the five bat diet classes, and all their references (Supplementary Materials). Finally, by examining the most recent decade’s publications, a top five list of the most-cited microorganisms was independently created for viruses, bacteria, fungi, and protozoa. The information was categorized according to bat dietary ecology (Figure 1) and distribution across continents (Figure 2).

## 3. Ecology and Microbiome of the Gastrointestine of *Chiroptera*

Recently, the microbiome’s definition has been revised to allow for a holistic view of microbial operation and interaction with the environment. The microbiome includes the microbiota and its “activity theatre” represented by structural elements and microbial metabolites, mobile genetic elements (including viruses) and surrounding environmental conditions [27]. The microbiota consists of a remarkable heterogeneity and quantity of microorganisms belonging to different prokaryotic and eukaryotic kingdoms that reside within the body and on the skin of the host to form a complex ecosystem in which the bacteria constitute the main part [27]. The virome instead is defined as the complex of all viruses of eukaryotic and prokaryotic cells found inside or on an organism [28,29].

Some studies have suggested that the host’s phylogeny plays the most important influence on the composition of the microbiome compared to other factors, including diet and environment [30,31], while other authors hypothesized that diet strongly influences the composition of the microbiome [32]. In other studies, it is suggested that the composition of the microbiome converges in relation to both diet and the host’s phylogeny [33].

Depending on their eating habits, *Chiroptera* can be divided into six distinct groups: (a) *Insectivorous*: The majority of *Chiroptera* have a basically insectivorous diet, catching their prey during flight; some of them can also feed on fruit. A population of bats within a large urban area can arrive to consume up to 14 tons of insects in a single night [34]. The smallest-sized bats belong to this group. Generally, in the metabolism of insectivorous bats, chitinase plays a vital role in the digestion of chitin from the exoskeleton of the consumed insects and the gut bacteria provide enough chitinase to meet their needs [35]. (b) *Frugivorous:* Frugivorous bats feed exclusively on fruit and some other plant parts. They often feed in groups and can travel long distances in search of food. Among them are the large flying foxes (*Pteropodidae*) [36], but also forms of much smaller sizes with wingspans not exceeding 30 cm. In these bats, the protein requirement is met by ingesting pollen and leaves [37,38]. Frugivorous bats, as with other animal species, rely on the cellulolytic and xylanolytic activities of the resident microorganisms to access to the leaf’s nutrients, thus strictly depending on the symbiotic bacteria hosted in their gut [6]. (c) *Nectarivorous:* Nectarivorous bats feed primarily on nectar and some insects collected from the flowers they visit. They are generally small and are characterized by a substantial modification of the muzzle and tongue, which is considerably elongated and provided with bristle-like papillae at the tip. Like the frugivorous forms, they are essentially inhabitants of tropical areas. The primary source of carbohydrates for nectarivorous and frugivorous bats is probably fruit, while their main protein source can be identified in leaves and flowers: the presence of gut bacteria such as *Enterobacter*, *Klebsiella*, and *Serratia* genera could also have a complementary role in carbohydrate digestion, favoring the breakdown of most sugars, including xylose, which is one of the main components of plants [36]. (d) *Hematophagous*: True vampire bats of the *Desmodontinae* subfamily feed on the blood of small animals such as birds or livestock, piercing the skin of their prey with their incisors. This practice can lead to the transmission of serious viral diseases to victims, such as rabies, or to infections of the wound. The common microbiota of vampire bats is composed mainly of phyla *Firmicutes*, *Proteobacteria*, *Tenericutes*, and *Epsilonbacteraeota*. Vampire bats have an intestinal microbiota that is compositionally distinct from that of other bats, as predicted by their exclusive diet of blood, which causes a deficiency in vitamins and lipids, as well as high wastage of nitrogen and challenges to osmotic homeostasis [37]. Most data refer to *Desmodus rotundus* and not much is known about the microbiome of other vampire bats [38]. (e) *Omnivorous*: Omnivorous bats feed on small mammals, including other bat species, birds, lizards, and frogs. They have a more varied diet, as they also feed on fruit and insects. The largest micro-*Chiroptera*, such as the false vampire (*Megadermatidae*) and the spectral bat (*Fillostomidae*), belong to this group. Based on feeding strategies, the microbiome differs significantly: the species diversity in the bacterial community showed an increase from fruit-eating bats to insectivorous and omnivorous bats, a trend opposite to that of the fungal community [35]; the blood microbiota of omnivorous bats is composed of several potentially pathogenic bacterial genera, such as *Bartonella* and *Mycoplasma*, and could vary depending on host ecological and physiological features. Furthermore, the relative abundance of microbial species in communities can differ depending on the bat’s food sources, which could influence the prevalence of bacterial genera in other physiological districts of omnivorous bats [39]. (f) *Piscivores*: Fish-eating bats can catch fish underwater or on its surface using the hooked claws at the tips of the toes of their hind limbs (*Noctilionidae*, and some species of *Myotis*). Some studies have shown that piscivorous bats carry a characteristic gut microbiome in which the most relevant bacteria are directly acquired from their preys. This characteristic microbiome shows an enrichment of genes involved in vitamin synthesis, as well as a complex carbohydrate and lipid metabolism, probably providing their hosts with a greater ability to metabolize glycosphingolipids and long-chain fatty acids that are particularly abundant in fish [3].

In summary, the feeding niche modulates the structure and ecological relationships of microbial communities [40] and in the case of the gut microbiome, diet variability is one of the main mechanisms influencing the stability of these communities [41]. For example, the type and concentration of nutrients determine the abundance and composition of some bacteria, fungi, and protozoa [42].

Microbiomes can also vary within the same bat population with age and sex, when certain needs, such as pregnancy or the achievement of fertility in males, push for such a change [43,44]. In many cases, the presence of a particular microbial package determines greater resistance to fungal parasites [45,46]. In recent studies, it has been seen how the eating habits of *Chiroptera* colonies can influence the composition of the antigenic repertoire of individuals, favoring or depressing the presence of microorganisms in the bloodstream [47,48]. Similarly to the gut microbiome, blood microbiome is influenced by the host genetic and immune characteristics, by the translocation of microorganisms from other organs, and by the availability of nutrients [49,50]. The latter depends on the absorption by intestinal cells, which modulate the type and concentration of biomolecules in the bloodstream, thus regulating the transport and distribution of nutrients [48]. Therefore, the feeding niche could regulate the structure, composition, and ecological relationships of the various microorganisms in the host’s bloodstream, working as one of the most important factors regulating the physiology and ecology of the host. Finally, the variability in microbial taxa can be significantly high when omnivorous species are compared with frugivorous or piscivorous species [51,52,53].

Gut bacterial community is influenced by the specific type of food consumed. Animal-based diets can shape the intestinal microbiome in a very different way, even for species of bats that generally have the same type of diet, for example, insectivorous [54].

Bat microbial pools include zoonotic viruses and bacteria, such as *Herpesvirus*, *Coronavirus* and *Picornavirus* (often in co-infection) [55], *Leptospira* [56], *Bartonella* [57], *Mycoplasma* [58], *Borrelia* [59], and *Coxiella* [36]. Moreover, various studies report the presence of fungi and protozoa, such as *Leishmania* [60,61], *Plasmodium* [62], and *Trypanosoma* [63,64,65,66,67,68]. Notably, few studies describe blood microbial communities in bats, as well as their implication as a source of transmission of zoonotic diseases and generally in pathogen spread [69].

Many studies have focused on specific body districts, such as skin, ocular mucous membrane, oral cavity, stomach, intestine, internal organs, and blood, in attempts to provide a broad view of microbiota–host interactions, and the relationships occurring between the presence of microbial species and susceptibility/resistance to disease [70,71]. Besides being relevant to control of zoonosis, a profound understanding of pathogens composing the bat microbiota is crucial to bats’ safety and well-being, according to a comprehensive One Health vision.

Several studies focusing on different *Chiroptera* species of suggest a positive correlation between ectoparasites, endoparasites, and viral load. These studies identified the genus *Macroglossus* as the richest in parasites and the genus *Rhinolophus* as the poorest while the species *Hipposideros armiger* has proven to be the richest in viral load and *Cynopterus sphinx* the poorest [72,73]. Habitats and distribution seem to play a central role in shaping microbiome and play a role in preserving bat colony health. Studies on *Rousettus* bats (*Pteropodidae*) of the Old World [74,75] report that the potential danger of pathogens transmission (including neglected ectoparasites) is directly proportional to habitat loss.

A hot topic in bat microbiota is the study of guano. Bat guano promotes the multiplication and spread of fungi, including pathogenic yeasts and dimorphic fungi known to cause severe endemic mycoses such as histoplasmosis and fatal cryptococcosis, which are particularly lethal in immunocompromised individuals [10]. Additionally, studies have revealed the presence of antibiotic-resistant bacteria (methicillin-resistant *Staphylococcus aureus* (MRSA), ESBL-producing, and colistin-resistant Enterobacterales) and viruses (*Parvoviridae*, *Circoviridae*, *Adenoviridae*, *Poxviridae*, *Picornaviridae*, *Astroviridae*, and *Coronaviridae*) in guano dropped by various insectivorous bat species [76,77,78,79].

Bacteriophages belonging to *Siphoviridae* and *Microviridae* and plant and fungal viruses (*Luteoviridae*, *Secoviridae*, *Tymoviridae*, *Partitiviridae*, and *Sobemovirus*) were identified in the same studies. Finally, D-Coronavirus has been isolated from guano samples of the common fruit bat (*Pteropus medius*) in Sri Lanka [77].

## 4. Microbiome and Zoonotic Potential

Under physiological conditions, the *Chiroptera* microbiome is mainly characterized by a pool of microorganisms living in symbiosis or in commensal relationships with their host without causing any type of disturbance in an enzootic cycle [80,81]. Habitat degradation and reduction, climate change, loss of biodiversity, and urbanization may act disrupting the natural relationships between wild and domestic sympatric animal species, including humans [82,83,84], favoring pathogen spill-over and zoonotic events.

Coevolving with a microbial community multiplying in a constant enzootic cycle, bats serve as reservoirs for different emerging zoonotic pathogens, as well as for many ethological agents of many humans and animals’ endemic diseases. Moreover, bats are sympatric to human and possess the ability to migrate over large geographical distances [85].

Under this context, a full understanding of bat microbiota, with particular focus on potential pathogens, is crucial to public health management. Table 1 shows a short summary of zoonotic diseases commonly transmitted by bats, as reported by Dhivahar and colleagues [6].

High-throughput sequencing technologies have proven useful for studying the diversity and dynamics of species that make up the microbiome, even in complex systems such as the intestine [36] or blood [86]. Despite the vast amount of next generation sequencing (NGS) data functional analyses of microbiota are still overlooked [87].

## 5. Bat Microbiota

The microbiota is a complex community of microorganisms that may be crucial in maintaining a healthy physiological, immunological, and reproductive environment. It is molded by host, environmental factors, and by their interplay. It can rapidly respond to environmental changes due to the adaptability or responsiveness of the microbial community, but also thanks to the brief generation time of microorganisms and their high mutation rate [88]. Researching how animals adjust their microbiota to distinct life stages and under severe environmental conditions provides valuable insights into microbiota influence on host biology [89] and on the interplay with dietary habits. According to literature search queries, Figure 1 and Figure 2 show the most frequently reported microorganisms according to bat dietary ecology and continent, respectively.

### 5.1. Bat Virome

The growing interest in the role of bats as zoonotic reservoirs and advances in molecular detection technologies has promoted efforts in investigating potential public and veterinary health risks, and multi-viral infections [55,90]. Bats can either act as reservoirs of zoonotic viruses or can host everchanging genetic pools promoting multiple spillover events, and eventually creating viral variants able to infect humans [24,91,92,93]. The recent emergence of SARS-CoV-2 and the related COVID-19 pandemic represents the most striking paradigm for bat-borne viruses [94,95,96,97]. Compared to other mammals, the ability of bats to adapt to a variety of deadly viruses makes them better multipathogen carriers capable of spreading infectious agents during their short-lasting illness (or even no illness) before overcoming infection [24]. The bat virome varies according to dietary ecology (Table 2, Figure 1A) and geographic location (Figure 2).

### 5.2. Bacteriome

Studies on bacterial microbiota provide a relatively complete overall picture. Indeed, studies targeted different districts, ranging from the oral cavity [98] to the ocular mucosa [70], stomach [53,99], intestine [42], kidneys [100], and blood [47,48]. According to dietary ecology, there is a certain degree of bacterial specificity, for example, in nectarivorous and frugivorous bats compared to hematophagous bats. Among New World bats, the phylogenetic diversity in intestinal bacteria has been found to be associated with dietary strategy, resulting higher in bats that feed on fruit and lower in bats that feed on blood [38,48]. These differences can be explained by the type of diet and/or the transfer of bacteria from ingested prey. For instance, in insectivorous saliva has significantly higher pH and better buffering capacity compared to frugivorous saliva, which could be explained as an evolutionary defense against potentially harmful microorganisms: these bats produce chitinase to metabolize chitin with enzymatic activity higher in the pH range of 5.0–6.0 [35]. It is known that various bacterial genera produce chitinase [101,102]. Although these bacteria are not exclusive to insectivorous, chitinase-producing bacteria have so far been found only in bats that feed on insects [101]. Moreover, very often, the richness and diversity in bats’ bacteriomes provide a valuable barrier against disease and permanent protection against sometimes destructive fungal infections [45,103,104]. Bacteria investigated in bats vary according to dietary ecology (Table 3, Figure 1B) and geographic location (Figure 2).

Despite the presence of *Aeromonas* in their intestine, piscivorous bats show low levels of aerolysin, probably through gene suppression or modulation in their intestine [3]. All of them can indiscriminately host coagulase-negative staphylococci and other Gram-positive bacteria, Leptospira, and *Listeria monocytogenes*. These infections are usually subclinical or asymptomatic.

### 5.3. Mycobiome

Bats, especially frugivorous ones, are carriers of various pathogenic fungi [23]. Next-Generation Sequencing studies have shown that the intestinal fungal community is significantly influenced by the host’s feeding habits, and particularly the intestinal mycobiota of frugivorous bats, which can be mainly composed of food-derived fungi. Bat mycobiomes vary according to dietary ecology (Table 4, Figure 1C) and geographic location (Figure 2).

Despite its local distribution and decline, *Pseudogymnoascus destructans*, the causative agent of white-nose syndrome [WNS] is responsible for the death of millions of bats in North America [105]. It is a psychrophilic fungus that infects the skin of bats during winter season while they are hibernating. The fungus can invade living tissues of the animal, causing characteristic severe skin lesions. The cutaneous microbiota of bats, strongly influenced by complex and interacting factors, can influence the growth of useful microorganisms, such as *Pseudomonas* and *Rhodococcus* detected on bat skin, which confers resistance to WNS [103].

### 5.4. Protozoa

Bats host numerous species of protozoa and serve as reservoirs for some of them. In humans, the main protozoan-associated diseases are malaria, leishmaniasis, toxoplasmosis, trypanosomiasis, and cryptosporidiosis. To date, no correlation has been observed between humans and bats in relation to malaria. Infections in humans are caused by six species of the *Plasmodium* genus of the Haemosporidia order [106]. However, many other hemosporidian malaria-related parasites are present in wild populations, including bats [107]. Information on the presence of *Toxoplasma gondii*, which infects several warm-blooded animal species, including humans, is still limited and has been obtained through the detection of antibodies and DNA of the microorganism [108].

Bats have also been incriminated as potential reservoirs of several *Leishmania* species such as *L. braziliensis*, *L. mexicana*, *L. infantum*, and *L. amazonensis* [61,109]. In various studies, these bacteria have been detected in the liver, spleen, and skin. Recently, *Leishmania* has also been detected in blood samples [61]. It has been observed that *Leishmania* infection rates in bats are higher in frugivorous bats. Various species of bats have been reported as hosts of *Trypanosoma* spp. [63,64,65,66,67,68]. For example, the presence of *Trypanosoma cruzi* has been reported in the saliva of four species of neotropical bats in northern Peru [66]: two of them were hematophagous bat species, and given the regional importance of Chagas disease, the authors emphasized the need for further research on the potential risk of zoonotic transmission directly from bat bites. *Trypanosoma cruzi* has also been detected in a migratory bat species in Oklahoma [64]. *Cryptosporidium* spp. and *Giardia duodenalis* are common etiological agents of diarrheal diseases in humans and animals worldwide [110,111]. In particular, the human pathogen *Cryptosporidium parvum* has been identified in two insectivorous bats from the United States and the Czech Republic [112]. In addition, the presence of the human-specific *Cryptosporidium hominis* has been reported in free-tailed bats in captivity in Australia [113]. Although the role of bats in the transmission of *Cryptosporidium* spp. to humans remains to be clarified, these results highlight the potential transmission of these microorganisms; however, current evidence suggests that bats are mainly infected with bat-specific genotypes and lack evidence of active infection of bats with zoonotic species of Cryptosporidium [114]. Finally, although giardiasis is a disease commonly reported in large number of mammals, including humans, little is known about the presence and prevalence of Giardia species in bats; therefore, screening and genotyping positive Giardia samples are essential to evaluate Giardia zoonotic risk [115]. Bat protozoa vary according to dietary ecology (Table 5, Figure 1D) and geographic location (Figure 2).

Insectivorous bats have a wide and complex behavioral and ecological range, which includes the choice of insect diet, water bodies, and sharing an ecosystem with other vertebrates that are essential for the transmission of certain parasites [116].

## 6. Final Considerations and Perspectives

The bat microbiome is shaped by different factors, related to environment, social interactions among individuals and colonies, and diet [117]. These factors force bats to become highly specialized for specific habitats, on the one hand influencing their potential as reservoirs of zoonoses, and on the other hand impairing their response to environmental changes [48]. According to literature queries, the bat microbiome varies depending on feeding ecology and geographical distribution (Figure 1 and Figure 2). While the link between microbiome composition and diet can be intuitive, it should be noticed that identification of pathogens in different continents is biased; local outbreaks often attract the attention of scientists, and this influences the microbial targets chosen for research and publication (e.g., Ebola in Africa or lyssavirus in Australia). Historically, human encroachment and loss of habitat increased human–bat sympatry, and this encouraged their incrimination as responsible for the emergence, spread, and diffusion of many emerging zoonotic diseases [24,93,118,119,120,121]. Furthermore, there is a discrepancy between the dissemination of information on the ecological role of bats and their role as disease-spreaders [25,122]. Bat discreditation promoted the hunting and persecution of bats, often resulting in colony extermination [123]. To protect bats, a treaty on the conservation of European bat populations [EUROBATS] was signed by 32 countries in 1994. Many bat species are currently included in the IUCN most endangered categories, and at least all *Pteropus* species in CITES Appendix II, due to illegal trade resulting from their use as a food source [124]. Recently, by developing BRT models, Guy and coworkers predicted that bat species that are better studied, longer-living, form larger social groups, and have larger geographic ranges east of the Prime Meridian carry the greatest number of viral families [90]. According to the same study, six bat species appear to have a higher likelihood of being viral zoonotic carriers: four insectivorous (*Asellia tridens*, *Barbastella barbastellus*, *Coelops frithii*, and *Myotis grisescens*), one omnivore (*Phyllostomus hastatus*), and one frugivore (*Pteropus rodricensis*). It should be noticed that the IUCN classifies *Asellia tridens* and *Coelops frithii* as least concern and *Pteropus rodricensis* as endangered; possible strategies to minimize zoonotic risk should include measures for the protection and conservation of bats and their habitats, from a modern and effective One Health perspective [84,125]. Indeed, besides being reservoirs for zoonoses, bats also play crucial roles in their habitats. Frugivorous/nectarivorous bats represent the only mean of pollination for some botanical species [126,127]. Insectivorous bats consume huge quantities of insects, which can play a role as vectors of animal and human diseases, or as pests [128,129]. Finally, bats are an excellent indicator for the health of ecotones and urban habitats [130], and a fundamental model for studies on longevity and immune resistance [13,50,131]. Bats adapted to different environments and feeding habits. Consequently, they are more susceptible and sensitive to habitat changes induced by human activities, climate change, and external and disturbing factors. The greater their specialization, the lower their degree of resilience, and this also affects the diversity in their microbiome [132]. The presence of specific bacterial pools contributes to host health and physiological balance by counteracting invasion by other bacteria and fungi [45,46] and subsequently limiting clinical outcomes to subclinical or asymptomatic [58,133]. A lower availability of food or a forced shift towards other food sources could disrupt the already precarious natural balance that many species, threatened or at risk of extinction, are a part of. Eventually, it would be useful to manage the ecotones resulting from urbanization and avoid the destruction of natural habitats, their trade, and deforestation [41,88,134,135], and to consider the crucial role of bats in various ecosystem services. This requires a One Health approach to fill knowledge gaps and ensure the management of mitigation strategies, not only to minimize the risk of zoonoses but also to ensure the conservation of these highly useful species [136,137,138,139].

## 7. Conclusions

Bat microbial communities may vary even in conspecific hosts and are influenced by host physiology, feeding behavior and diet, social interactions, but also by habitat diversity and climate change. A growing number of studies suggest that animal microbiota may respond in various ways to changes in land use, particularly when such changes lead to altered or deficient food resources. From a conservation perspective, understanding the potentially negative and indirect effects of habitat destruction on animal microbiota can also play a crucial role in the conservation and management of the host itself. According to the One Health concept, which recognizes an interdependence between humans, animals, and the environment, the bat microbiota represents an indicator of host and environmental health, besides allowing for evaluation of the risk of emerging infectious diseases.

Data reported in the literature about the bat microbiome focus almost entirely on the discovery of microbial species potentially pathogenic to other animal species and to humans. This represents the first level of investigation of microbial communities associated with different anatomical districts. However, the exact role of individual species and their contribution to pathogenicity and immune responses in potential new hosts and reservoirs is still lacking. Furthermore, deep comprehension of the impacts of microbial communities and pathogens in animal conservation, as well as in veterinary and public health, is still missing. Future studies should focus on filling these gaps and provide deeper knowledge on the composition and functional analysis of bat microbiomes. This information is paramount to implement correct habitat and host management and to develop effective surveillance protocols worldwide.

## Figures and Tables

**Table 1:** Etiological agents commonly reported in bats and related zoonotic diseases.

	Etiological Agent	Disease
**Bacteria**	*Bartonella* spp.	Bartonellosis
	*Pasteurella* spp.	Pasteurellosis
	*Borrelia* spp.	Borreliosis or Lyme disease
	*Leptospira* spp.	Leptospirosis
	*Aeromonas hydrophila*	Bloodstream infection
	*Rickettsia* spp.	Rickettsiosis
	*Salmonella typhi*	Typhoid fever
**Fungi**	*Histoplasma capsulatum*	Histoplasmosis
	*Coccidioides* spp.	Coccidioidomycosis or valley fever
	*Candida* spp.	Candidiasis
	*Cryptococcus* spp.	Cryptococcal diseases including meningitis
**Protozoa**	*Babesia* spp.	Babesiosis
	*Entamoeba histolytica*	Amoebiasis
	*Trypanosoma cruzi*	Chagas disease
	*Plasmodium* spp.	Malaria
	*Cryptosporidium* spp.	Cryptosporidiosis
	*Leishmania* spp.	Leishmaniasis
	*Toxoplasma* spp.	Toxoplasmosis
	*Giardia* spp.	Giardiasis
**Viruses**	*Henipavirus hendraense*	Paramyxovirosis
	*Henipavirus nipahense*	Paramyxovirosis
	SARS coronavirus, SARS-CoV-2	Coronavirosis
	MERS coronavirus	Beta-coronavirosis
	Ebola virus and Marburg virus	Filovirosis

**Table 2:** Selected viruses according to bat feeding ecology.

	Insectivorous	Frugivorous/Nectarivorous	Piscivorous	Hematophagous	Omnivorous
*Adenoviridae*	•			•	
*Alfainfluenzavirus*				•	
*Alphavirus*		•			
*Astrovirus*		•			
*Bacteriophage*				•	
*Betainfluenzavirus*		•			
*Betapapillomavirus*		•			
*Bunyaviridae*	•			•	
*Caliciviridae*					
*Chordopoxvirinae*				•	
*Circoviridae*	•				
*Coronaviridae*	•		•		•
*Equine encephalitis virus*			•		
*Filoviridae*	•				
*Gammaretrovirus*		•			
*Hepatovirus*		•			
*Herpesviridae*	•			•	
*Lloviu Virus*	•				
*Lyssavirus*	•	•	•	•	
*Mammarenavirus*		•			
*Mastadenovirus*		•		•	
*Metapneumovirus*		•			
*Morbillivirus*					•
*Nairoviridae*	•				
*Orthopoxvirus*		•			
*Paramyxoviridae*			•		
*Paramyxovirus*	•				
*Picornaviridae*	•				
*Picornaviridae*	•			•	
*Porcine endogenous retrovirus*				•	
*Roseolovirus*		•			
*Rotavirus*	•	•			
*Totivirus-like*	•				
*a*, *b-Coronaviridae*	•	•			

**Table 3:** Selected bacteria reported in bats according to feeding ecology.

	Insectivorous	Frugivorous/ Nectarivorous	Piscivorous	Hematophagous	Omnivorous
*Acidobacteriota*					*•*
*Aeromonas*			*•*		
*Aeromonas hydrophila*				*•*	
*Anaplasma*					*•*
*Anaplasma phagocytophilum*	*•*				
*Bacillus*	*•*				
*Bacillus cereus*		*•*			
*Bacteroidota*					*•*
*Bartonella*	*•*			*•*	*•*
*Burkholderia*	*•*				
*Campylobacter coli*		*•*			
*Campylobacter jejuni*		*•*			
*Campylobacterota*					*•*
*Cetobacterium*			*•*		
*Citrobacter*	*•*				
*Ehrlichia*					*•*
*Enterobacter*		*•*			
*Enterococcus*	*•*	*•*			
*Enterococcus faecalis*		*•*			
*Escherichia*		*•*			
*Firmicutes*		*•*			*•*
*Fructobacillus*		*•*			
*Gemella*					*•*
*Helicobacter*	*•*				
*Hhemoplasms*				*•*	
*Klebsiella*		*•*			
*Lactobacillus*	*•*				
*Lactococcus*	*•*				
*Mycoplasma*					*•*
*Neisseriaceae*	*•*				
*Neorickettsia*					*•*
*Paeniclostridium*	*•*				
*Pantoea agglomerans*		*•*			
*Paraclostridium*			*•*		
*Pasteurellaceae*	*•*				
*Photobacterium*			*•*		
*Plesiomonas*	*•*		*•*		
*Pseudomonas aeruginosa*		*•*			
*Staphylococcacee*		*•*			
*Staphylococcus aureus*		*•*			
*Staphylococcus saprophyticus*		*•*			
*Tenericutes*		*•*			
*Undibacterium*	*•*				
*Ureaplasma*		*•*			
*Weissella*		*•*			
*Yersiniaceae*		*•*			

**Table 4:** Selected fungi reported in bats according to their feeding ecology.

	Insectivorous	Frugivorous/Nectarivorous	Piscivorous	Hematophagous	Omnivorous
*Ajellomycetaceae*				•	
*Alternaria alternata*				•	
*Amycolatopsis mediterranei*				•	
*Ascomycota*		•	•		
*Aspergillus flavus*				•	
*Aspergillus* spp.	•				•
*Basidiomycota*		•	•		
*Candida albicans*					•
*Candida glabrata*					•
*Candida parapsilosis*					•
*Candida* spp.	•	•			
*Cryptococcus* spp.	•				•
*Cutaneotrichosporon moniliiforme*	•				
*Debaryomyces hansenii*	•				
*Debaryomyces* spp.	•				
*Fusarium* spp.	•				
*Histoplasma capsulatum*	•				
*Penicillium* spp.					•
*Pneumocystis*	•				
*Scopulariopsis* spp.					•

**Table 5:** Most common protozoa reported in bats according to their feeding ecology.

	Insectivorous	Frugivorous/Nectarivorous	Piscivorous	Hematophagous	Omnivorous
*Acanthamoeba castellanii*				•	
*Cryptosporidiidae*				•	
*Cryptosporidium* spp.	•				
*Cryptosporidium* spp.			•		•
*Eimeria* spp.	•	•			
*Entamoeba* spp.	•	•			
*Giardia* spp.	•				•
*Isospora* spp.	•				
*Sarcocystis glareoli*					•

## Data Availability

No new data were created or analyzed in this study. Data sharing is not applicable to this article.

## Supplementary Materials

The following supporting information can be downloaded at: https://www.mdpi.com/article/10.3390/ani14203043/s1, Table S1: Database.
